# Preferences for Sun Protection With a Self-Monitoring App: Protocol of a Discrete Choice Experiment Study

**DOI:** 10.2196/16087

**Published:** 2020-02-08

**Authors:** Vasileios Nittas, Margot Mütsch, Milo Alan Puhan

**Affiliations:** 1 Epidemiology, Biostatistics and Prevention Institute University of Zurich Zurich Switzerland

**Keywords:** preventive medicine, mobile health, health informatics, health economics, patient preferences, discrete choice experiment

## Abstract

**Background:**

The incidence of sun-exposure-related skin conditions, such as melanoma, is a gradually increasing and largely preventable public health problem. Simultaneously, the availability of mobile apps that enable the self-monitoring of health behavior and outcomes is ever increasing. Inevitably, recent years have seen an emerging volume of electronic patient-generated health data (PGHD), as well as their targeted application across primary prevention areas, including sun protection and skin health. Despite their preventive potential, the actual impact of these apps relies on the engagement of health care consumers, who are primarily responsible for recording, sharing, and using their PGHD. Exploring preferences is a key step toward facilitating consumer engagement and ultimately realizing their potential.

**Objective:**

This paper describes an ongoing research project that aims to elicit the preferences of health care consumers for sun protection via app-based self-monitoring.

**Methods:**

A discrete choice experiment (DCE) will be conducted to explore how healthy consumers choose between two alternative preventive self-monitoring apps. DCE development and attribute selection were built on extensive qualitative work, consisting of the secondary use of a previously conducted scoping review, a rapid review of reviews, 13 expert interviews, and 12 health care consumer interviews, the results of which are reported in this paper. Following D-optimality criteria, a fractional factorial survey design was generated. The final DCE will be administered in the waiting room of a travel clinic, targeting a sample of 200 participants. Choice data will be analyzed with conditional logit and multinomial logit models, accounting for individual participant characteristics.

**Results:**

An ethics approval was waived by the Ethics Committee Zurich. The study started in September 2019 and estimated data collection and completion is set for January 2020. Five two-level attributes have been selected for inclusion in the DCE, addressing (1) data generation methods, (2) privacy control, (3) data sharing with general practitioner, (4) reminder timing, and (5) costs. Data synthesis, analysis, and reporting are planned for January and February 2020. Results are expected to be submitted for publication by February 2020.

**Conclusions:**

Our results will target technology developers, health care providers, and policy makers, potentially offering some guidance on how to design or use sun-protection-focused self-monitoring apps in ways that are responsive to consumer preferences. Preferences are ultimately linked to engagement and motivation, which are key elements for the uptake and success of digital health. Our findings will inform the design of person-centered apps, while also inspiring future preference-eliciting research in the field of emerging and complex eHealth services.

**International Registered Report Identifier (IRRID):**

PRR1-10.2196/16087

## Introduction

### Background

As the mHealth market rapidly expands, digitally self-monitoring our health and well-being is easier than ever before. Inevitably, the volume of available electronic patient-generated health data (PGHD) grows exponentially. Defined as nonclinical health information, generated and controlled by consumers, patients, and their designees, PGHD are widely used across public health domains to facilitate primary prevention and strengthen health promotion [1-4]. Mobile phones and wearables come as fully functional measurement devices, accompanied by an abundance of apps that collect PGHD and provide prevention-relevant feedback [2,5]. Many of those apps are capable of capturing physical and contextual signals, as well as communicating risks and supporting behavior change [6]. With such an unprecedented number of self-monitoring apps comes an equally unprecedented need to understand how these should be designed and utilized for successful primary prevention.

### Mobile Self-Monitoring for Sun Protection

Serious skin conditions related to sun exposure, such as melanoma, are on the rise. Melanoma—one form of skin cancer—is a potentially fatal malignancy of the skin arising from atypical melanocytes, primarily affecting young and middle-aged population groups [7,8]. While disease onset depends on multiple factors (eg, family history and genetics), exposure to UV light (eg, sun and indoor tanning) is considered a primary risk factor [7]. The global incidence of melanoma indicates upward trends, with most rapid increases recorded in western and Caucasian populations [7]. While the epidemiological trends of melanoma indicate a very present and most likely growing public health problem, targeted behavioral change in relation to sun protection can mitigate much of its burden [7]. With increasing popularity of mobile self-monitoring across prevention areas, including weight loss, physical activity, nutrition, smoking, alcohol consumption, and mental health, the use of PGHD is gradually gaining popularity in sun protection [1-4,9-13]. Mobile apps are designed to monitor behavior (eg, sunbathing intensity, use of sunscreen, and use of protective clothing), as well as environmental exposure (eg, UV-light intensity), and to combine that with behavior change techniques, such as tailored messages, sensitive reminders, motivational feedback, gamification, and education [11-13]. Acknowledging the need for person-sensitive and personalized primary prevention, the emergence of mobile self-monitoring is a unique opportunity and resource in reducing sun-related skin conditions, such as melanoma and other skin cancers.

### An Emphasis on Health Care Consumer Preferences

A prerequisite of digital and mobile self-monitoring is the motivation of consumers to engage with technology. This is driven by individual, technical, social, and environmental factors, such as personal motivation, appropriate use, long-term engagement, and satisfaction [14,15]. While existing theories identify overall drivers of motivations of technology engagement, consumer preferences regarding concrete characteristics of technology have been less explored [16,17]. When it comes to one’s health and well-being, as well as the prevention of malaise (ie, discomfort), focusing on health care consumer preferences is a central component of person-centered care. Person-centeredness requires a full focus on the needs, values, and desires of individuals, as well as their environments and social contexts [18,19]. Evidence suggests that person-centeredness can enhance satisfaction with, and acceptance of, health services while ensuring engagement and adherence [20]. Understanding preferences and their predictors is key to developing acceptable self-monitoring technologies.

This study outlines the methodology and preparatory qualitative results of a discrete choice experiment (DCE) that aims to elicit consumer preferences for facilitating sun protection with self-monitoring apps. Our findings target health app providers, practitioners, citizens, and policy makers, aiming to guide a more preference-sensitive development and use of self-monitoring apps.

### Aims

Our study aims to assess the relative importance of modifiable elements of self-monitoring apps that focus on sun protection. We have identified the following study objectives:

1. Identify and explore which elements of self-monitoring apps are deemed important by health care experts and health care consumers (qualitative results).
2. Among those preidentified elements, elicit the relative importance of health care consumer preferences (DCE results).
3. Determine whether those preferences vary across participant characteristics, including age, gender, education, health app attitudes, and perceived health (DCE results).

## Methods

### Overview of Approach

**Figure 1:**
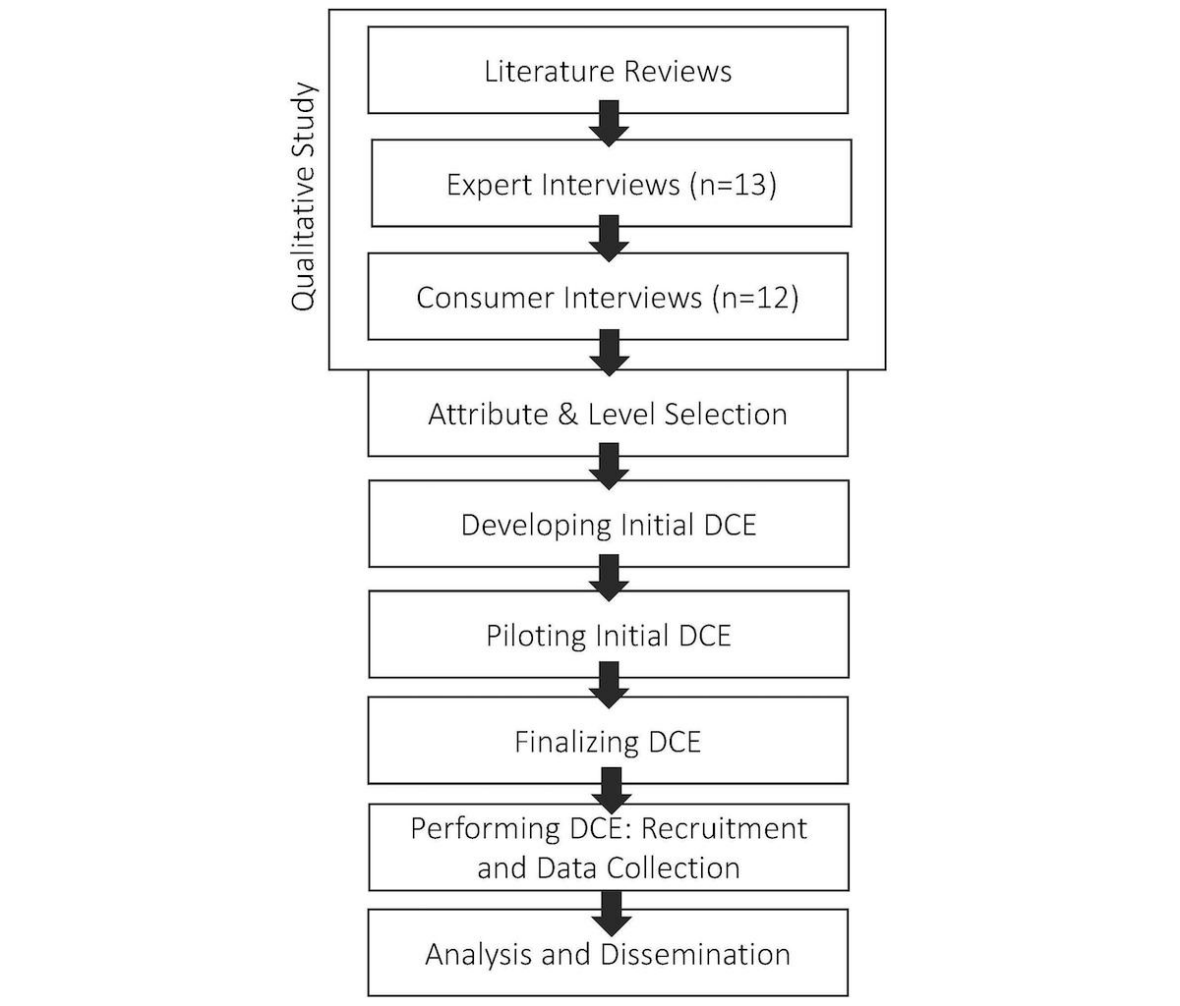
Overview of the study’s methodology. DCE: discrete choice experiment.

A DCE is a robust survey-based methodology that enables the elicitation of consumer preferences [21]. Rooted in psychometrics and based on strong theoretical grounds, DCEs have been widely used in economic research and are rapidly gaining popularity within health care [21-23]. The technique’s core assumption suggests that any good or service consists of distinguishable characteristics, also known as attributes, from which consumers derive utility [21,24]. Each attribute can take alternative forms, often described as levels. The derived utility varies with changing levels of these attributes. Individual choices among alternatives of these characteristics are assumed to indicate a person’s preferences, underlying values, and perceived service utility [21,24]. Developing a DCE and selecting appropriate attributes requires a range of preparatory qualitative steps [25]. We conducted literature reviews and interviews with health care consumers and experts, the results of which are detailed in this study [22]. Figure 1 provides an overview of the study’s methodological steps.

### Rationale for Using a Discrete Choice Experiment

Both mobile self-monitoring and primary prevention rely on engaged health care consumers. Understanding their preferences is, therefore, key to the successful development and use of self-monitoring technology for preventive purposes. DCEs enable the identification of those relative preferences by asking consumers to choose between at least two versions (ie, scenarios) of a good or service, each consisting of different bundles of attribute levels [21,26]. Respondents are requested to make repeated choices, which provides enough information to statistically elicit those elements that are perceived to yield the highest utility [26].

In having to choose one scenario over another, thus, being requested to make trade-off choices, DCEs provide strong indices of preferences and are gradually gaining popularity in eHealth research [21]. For example, Cranen and colleagues used the methodology to elicit preferences of chronically ill patients regarding telerehabilitation, exploring attributes such as physician communication modes, feedback provision, and the use of digital monitoring tools [27]. Similarly, Kaambwa and colleagues applied a DCE to investigate the telehealth preferences of the elderly, identifying an inclination toward comprehensive and inexpensive eHealth services that target those who face constrained access to traditional care [28]. Using DCEs will enable us to identify which attributes of prevention-focused self-monitoring apps are considered important, as well as how consumer preferences are distributed across them [26]. DCEs require a thorough and well-conducted qualitative basis, which enables the selection of correct and appropriate attributes. The qualitative work and its results are presented in the following paragraphs.

### Discrete Choice Experiment Scenario

Each DCE is framed around a hypothetical scenario that should be relevant to the targeted topic and specific enough to allow participants to make their choices accurately. For this DCE, each participant will be asked to imagine a mobile prevention app that targets sun protection and skin health by collecting information on the duration and intensity of sun exposure, followed by educational reminders on when and how to protect.

### Discrete Choice Experiment Development: Methods of Qualitative Preparatory Work

Prior to developing the DCE, we completed a thorough three-step qualitative study, using existing literature and stakeholder input to identify and select key attributes. We (1) used the output of a previous scoping review, (2) conducted a rapid review of systematic reviews on the use of electronic PGHD for primary prevention, (3) conducted 13 semistructured expert interviews, and (4) conducted 12 health care consumer interviews [29]. The literature reviews and expert interviews were merely meant to provide a preliminary basis of potential attributes and, therefore, had a broader scope on electronic self-monitoring for primary prevention. The health care consumer interviews were framed around sun protection and skin health, allowing us to identify attributes that are context specific.

### Literature Reviews

Both reviews aimed at mapping current evidence on the use of electronic PGHD for prevention and health promotion, as well as associated barriers and facilitators. The previously conducted scoping review entailed searches in seven databases, complemented by multiple additional and grey literature searches, yielding 183 eligible primary studies [30]. The rapid review was conducted in two databases—PubMed and the Cochrane Database of Systematic Reviews—and was limited to systematic reviews, yielding 13 eligible studies. Data extraction was based on predefined templates and analysis was thematic, with raw data being thematically clustered and mapped. Multimedia Appendix 1 provides the rapid review’s Preferred Reporting Items for Systematic Reviews and Meta-Analyses (PRISMA) flowchart, inclusion criteria, information on data extraction, and a list of all included studies [31].

### Expert Interviews

The 13 semistructured expert interviews were conducted between March 12 and April 4, 2019, either face-to-face or via Skype. They aimed to expand on and validate the list of attributes identified in the literature. Our expert selection criterion entailed that after completion of the interviews, each of the following areas should be the expertise of at least one interviewee: eHealth research, self-monitoring, digital prevention, data science, eHealth and data ethics, primary prevention, clinical practice, and citizen science. The number of interviews was not prespecified but continued until saturation was reached. Interviews were guided by semistructured questions and a list of fixed topics that had to be addressed. Informed by the literature reviews, those topics included the following: (1) barriers and facilitators of digital self-monitoring for primary prevention, (2) the technical aspects of these barriers and facilitators, and (3) the broader components of self-monitoring-based primary prevention interventions, such as the use of behavior change techniques. In addition, experts were provided with a list of 22 attributes identified in the reviews and asked to comment on them, mention potentially missing ones, and expand on those perceived as highly important. Attributes were categorized according to the three above-mentioned (1-3) or newly emerging themes. All experts provided verbal consent for researchers to audiotape, transcribe, and analyze the interviews. Recordings were deleted after transcription, without linkages to any personal information.

### Health Care Consumer Interviews

The 12 semistructured consumer interviews were conducted in Zurich, Switzerland, between May 15 and May 24, 2019. They aimed to capture which attributes of self-monitoring apps for sun protection are perceived as most relevant by health care consumers. Eligibility required a minimum age of 18 years and no chronic conditions. Participants were recruited at the University of Zurich Travel Clinic and selected purposively to ensure age and gender balance. Interviews were guided by semistructured questions and a list of fixed topics that had to be addressed, including (1) barriers and facilitators of self-monitoring for sun protection and skin health promotion and (2) all attributes that were identified in the reviews and expert interviews. In the interview’s first part, participants were asked to discuss what would encourage or discourage them to electronically collect their health data for sun protection purposes. The second part consisted of a Likert-scale rating of a list of attributes that were identified by the literature reviews and expert interviews. Each interview required approximately 20 minutes and all participants provided prior written informed consent, including a confirmation of all eligibility criteria. All contact and personal identification information required for recruitment and invitation was deleted immediately after completion of the interviews and replaced by unique ID numbers. Multimedia Appendix 2 provides the interview schedule, participant demographics (eg, age and sex), and selected interview quotes.

### Analysis of Expert and Health Care Consumer Interviews

All interviews were transcribed and analyzed with MAXQDA, version 18.2.0 (VERBI Software) [32]. Our analysis followed a hybrid approach of inductive and deductive coding [33]. Initial deductive coding was based on the above-mentioned fixed topics that guided the interviews, followed by an inductive, data-driven generation of new codes and their connection to subthemes and overarching themes. To ensure that our codes were understandable and complete, a random sample of three interviews was provided to an external coder who was instructed to use our code system and analyze the interviews independently. The coded interviews were compared and inconsistencies discussed and resolved. This led to a wording change for two codes, as well as the merging of two codes that were not distinguishable.

### Discrete Choice Experiment Development: Results of Qualitative Preparatory Work

Combined, the literature reviews and 13 expert interviews yielded a list of attributes that were categorized into six groups, including (1) *effort and support*, (2) *trust and control*, (3) *data sharing*, (4) *technology and design*, (5) *prevention-related content*, and (6) *incentives and disincentives*. A more detailed account of these categories is provided in Multimedia Appendix 3.

The 12 health care consumer interviews revealed six overall attributes that were perceived as important to the use of self-monitoring apps for sun protection; these included (1) *costs*, (2) *privacy and trust*, (3) *added value*, (4) *time and effort*, (5) *user-friendliness*, and (6) *incentives*. A more detailed account is provided in Multimedia Appendix 3.

### Selection of Attributes and Levels

**Figure 2:**
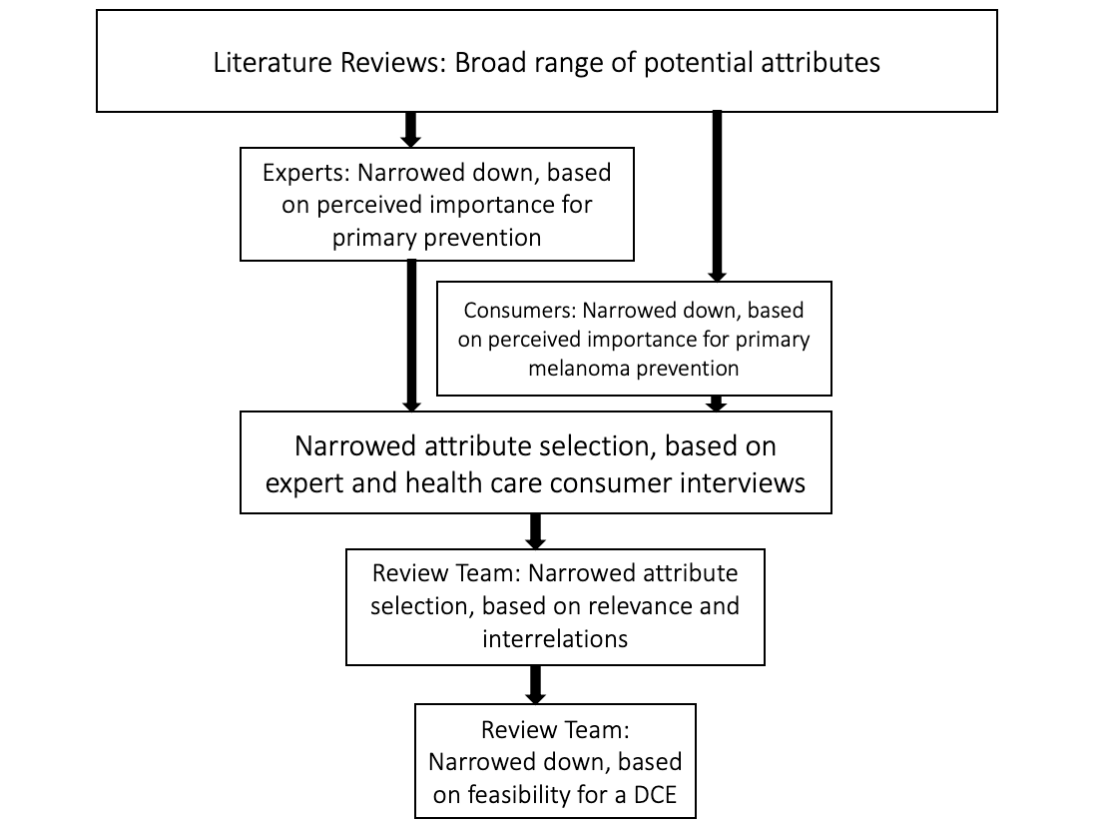
Attribute selection process. DCE: discrete choice experiment.

The final list of all possible attributes, resulting from a synthesis of review and interview findings, was reviewed by the research team and one consulted health care consumer. The selection was based on three overall criteria, as outlined by Bridges and colleagues, including (1) research question relevance, (2) decision context relevance, and (3) interrelations between attributes. The group’s choice was additionally guided by the importance of attributes, primarily by health care consumers, as well as by whether each attribute was realistic and could be defined for a DCE. We considered a realistic attribute to be compliant with current legal and policy regulations as well as with existing technology and primary prevention services. Consensus was reached on the following five attributes: (1) method of data generation, (2) privacy control, (3) data sharing with the general practitioner, (4) reminder timing, and (5) costs. The process and flow of attribute selection are provided in Figure 2.

We continued with assigning each attribute to realistic, relatable, and understandable levels. As suggested by Bridges and colleagues, we avoided ambiguous wording and tried to keep the number of levels to a minimum [22]. All attributes, levels, and descriptions were formulated in German and by native speakers. The wording has been reviewed by a native German speaker with a nonhealth background, ensuring ease of understanding and clarity. All included attributes and assigned levels are listed in Table 1.

We will additionally collect information on age, gender, highest attained education, attitudes toward health apps, and perceived health, as all of those factors have been previously associated with eHealth usage [34-37]. Perceived health will be measured with a widely used single-item measure, asking participants to rate their current general health on a 5-point scale from *very good* to *poor* [38,39]. Attitude will be captured with one reworded item on perceived usefulness, which was derived from previous research and asks participants to indicate their agreement on whether health apps are useful in promoting their health; they will rate their agreement on a scale from *totally agree* to *totally disagree* [40,41]. We chose single-item questions to keep that part of the questionnaire short and simple, considering that the actual DCE will require higher cognitive and time resources.

**Table 1:** Identified attributes, attribute levels, and prior assumptions.

Attributes	Descriptions	Attribute levels	Prior assumptions
1. Data generation method	How would you like your data to be collected?	(a) no manual entry (b) manual entry once a day	It is expected that most will prefer no manual data entry, as that is linked to lower effort. However, those who are more privacy-concerned might tend toward manual data entry.
2. Privacy control	If your data are being shared with third-party commercial entities, how would you like to control when and with whom your data are shared?	(a) I will only receive information on potential data sharing with third parties once and will be asked to provide informed consent once (b) I will be informed and provide consent whenever my data are provided to third parties	A clear a priori expectation is difficult to be formulated. Those who are concerned about constant push messages will likely prefer (a), and those who are more privacy-concerned will most likely choose (b).
3. Data sharing with general practitioner	Would you like to share the data collected with your general practitioner, to be discussed at your next visit?	(a) yes (b) no	A clear a priori expectation is difficult to formulate. We expect that those who have a trusting general practitioner relationship and lower perceived health will prefer (a).
4. Reminder timing	How would you prefer the times and frequency of your reminders to be set?	(a) I set the time and frequency of my reminders myself (b) the app sets the times and frequency of reminders automatically, based on my data	It is expected that most will prefer setting the time and frequency of reminders themselves, to avoid nuisance.
5. Costs	Are there any costs associated with downloading the app and, if yes, how high are these?	(a) free (b) one-time payment of 3 Swiss Francs	It is expected that most will prefer a free app. However, the cost might be accepted if combined with other desired attributes, such as low effort and high privacy.

### Experimental Design and Choice Sets Selection

The combination of included attributes and levels, as shown in Table 1, results in a full factorial design of 2^5^=32 possible distinct choice sets [42]. Limited time and cognitive capacities deem such a large survey design unrealistic. In our context, the time factor is particularly constraining, as questionnaires will have to be answered in the waiting room, often during short preconsultation windows. Although full factorial designs hold desirable features, such as perfect orthogonality and balance, it is common practice to use only subsets of those, known as fractional factorial designs. Thus, we developed a fractional factorial design, following D-optimality criteria and using R, version 3.5.3 (The R Foundation), the open-source software for statistical computing [43]. The 32 choice sets were reduced to a fractional factorial sample of eight. The quality of responses will be assessed through the inclusion of one additional choice set that will be identical to a previous one. In line with the axiom of completeness, participants that provide consistent answers are expected to choose the same alternative twice [22,44]. We will calculate percentages of inconsistent responses and assess their distribution across individual participant characteristics, as excluding participants is not recommended [22,45]. The final questionnaire version will include eight original and one repeated-choice set, yielding a total of nine choice sets.

### Discrete Choice Experiment Piloting and Validity

The survey was piloted face-to-face with 8 participants recruited from the University of Zurich Travel Clinic, ensuring understandability and feasibility. Participants provided written informed consent and received written detailed information on the study’s purpose, the research question, and all attributes. Participants were asked to complete the questionnaire in a think-aloud manner. The DCE’s face validity was tested through a discussion on the survey’s understandability and relevance, as well as on perceived ease of answering. Participants were asked to provide input on the survey’s wording, content, and design, including the provided background material, pictures, pictographs, and choice of colors. Particular attention was given to the perceived relevance, formulation, and understandability of the sun protection scenario. Time to completion was measured to ensure that the survey is feasible within a given time frame. We additionally assessed whether overall results are in line with our hypotheses (see Table 1) [46]. The pilot was followed by subsequent DCE adjustments, ensuring that the final questionnaire is easy to comprehend and complete.

### Participant Sampling, Recruitment, and Survey Administration

Estimating an adequate DCE sample size is lacking scientific consensus and remains a largely complex decision. Sample size decisions ultimately depend on multiple factors, such as task complexity, available resources, the sample’s composition, and the target statistical precision of findings [22,47,48]. Although parametric approaches have been proposed, when it comes to identifying minimum sample sizes for specific hypotheses testing, they are considered unsuitable [48-50]. That leaves many researchers to use rule-of-thumb-based estimations [50]. Examples of those range from an overall sample of 100-300 participants to a minimum of 20 participants per choice set [47,50]. Carefully considering available time and financial resources, we utilized the rule of thumb proposed by Johnson and Orme, which depends on the number of choice tasks, alternatives, and analysis cells [51,52]. Aiming for a large enough sample size to identify the main effects and interactions, we will target a sample of 200 participants.

Participants will be recruited in the waiting room of the University of Zurich walk-in Travel Clinic. The clinic constitutes a hub for pretravel preventive consultation, as well as general preventive services, including vaccinations. The approximately 20,000 annual consultations render the travel clinic an ideal recruitment site. During recruitment days, everyone entering the clinic will be informed about the possibility to participate. Interested volunteers will be shortly briefed by a team member on the study’s topic, purpose, and methodology; all this information will be additionally provided in written form. The completion of a DCE often poses high cognitive demands, for which the first pages will solely serve the purpose of preparing participants to answer the questionnaire. These first pages include information on (1) the study aims, (2) the study topic and key concepts, (3) detailed descriptions of each attribute, complemented by pictographs, and (4) instructions on how a DCE is filled out. Participants will have to confirm that all eligibility criteria are fulfilled, including a minimum age of 18 years, no chronic conditions, and mobile phone ownership. As the study does not include a follow-up session or any identifiable personal information, a signed participant informed consent form is not required. The survey will be administered in paper form and completed in a quiet room within the clinic. During recruitment and survey administration, a member of the staff will be present to answer questions and resolve uncertainties. We expect a survey completion time of about 10-15 minutes.

### Analysis Plan

The DCE’s main end points are the individual preferences of our participants, defined via the chosen attributes and their levels. Those will be assessed using a conditional logit model. This allows for the estimation of the relative importance of each attribute over the remaining ones, using the retrieved mean preference weights, as given by model coefficients [53]. To achieve this, our analysis will assess changes in weights within attributes—when changing from level (a) to level (b)—and the relative sizes of those across attributes [53]. The conditional logit model treats our findings as a function of the choice alternatives. It was developed by McFadden in 1973 and has been proven to be in accordance with the random utility theory, dividing a respondent’s utility into a systematic and a random element [53,54]. We will use the R package *support.CEs* to convert our dataset to a form that is suitable for analysis [55].

We will additionally explore our data with mixed multinomial logit (MMNL) models, treating our findings as a function of choice alternatives and individual participant characteristics. Expecting some preference heterogeneity, we chose an MMNL model over a multinomial logit model, as the addition of the error term can adjust for unobserved heterogeneity and adds to the generalization of results [51]. In contrast to conditional logit modeling, mixed logit models provide preference-weight estimates and standard deviations of those, based on the assumption of an underlying distribution of preference weights and, thus, capturing preference heterogeneity among participants [53].

We will additionally assess the amount of missing data. If the percentage is considerable, we will use multiple imputation using chained equations, using the R package *mice*, assessing the impact of missingness on our findings. Goodness of fit will be assessed by looking at the distribution of the residuals and by calculating McFadden’s pseudo R-squared [53]. Exploring variation, our analysis will test several individual characteristics for inclusion in our models, including age, gender, education, health app attitudes, and perceived health.

## Results

An ethics approval was requested by the Ethics Committee Zurich and was waived since this study does not fall under the Swiss human research law, which only applies to clinical studies that involve a certain level of risk, as well as the collection of sensitive health data. The study began in September 2019, and estimated data collection completion is set for January 2020. Data synthesis, analysis, and reporting are planned for January and February 2020. Results are expected to be submitted for publication by February 2020.

## Discussion

To the best of our knowledge, this is the first DCE that will explore health care consumer preferences for sun protection with self-monitoring apps. Our results will target technology developers, health care providers, and policy makers, potentially offering some guidance on how to design or use self-monitoring apps in ways that are responsive to consumer preferences and, thus, likely to maximize their engagement. Following good practice, our methodology is based on extensive and carefully designed qualitative work, ensuring that all included attributes are relevant and relatable. Nonetheless, the inclusion of all potentially relevant attributes is practically impossible in a DCE, as its feasibility depends on the required cognitive workload, as imposed by the number and nature of selected attributes. These should ideally be practical and limited to the most essential ones. This requires a careful and reasoned reduction process to allow for a small and feasible number of included attributes. Inevitably, this process includes trade-offs and the exclusion of attributes that might be relevant for a considerable proportion of the target population.

While DCEs constitute a robust and well-accepted approach for preference exploration, their focus on a limited number of variables inherently limits their capacity to capture broader factors that influence preferences toward sun-protection-focused self-monitoring apps. To fully understand the topic, our findings need to be followed up by qualitative and mixed-method research that will focus on understanding the individual and contextual factors contributing to certain preferences. Finally, as data collection will occur at the University of Zurich Travel Clinic and is subject to certain participant inclusion criteria, the data may not be fully generalizable to the entire Swiss or European population.

Despite these limitations, the attributes we have identified cover a considerable range of self-monitoring app characteristics, which are modifiable and, thus, adjustable to health care consumer preferences. Data collection effort, privacy, the flow of information, the sensitivity of push messages, and costs are all topics that are well-discussed in the literature and perceived as key by health care consumers and experts, which signals their potential to enhance the impact of self-monitoring apps. Preferences are ultimately linked to engagement and motivation, which are key elements for the uptake and success of any digital health approach. Ultimately, our work and findings will inform the design of person-centered self-monitoring apps for sun protection, while also inspiring future preference-eliciting research in the field of emerging and complex eHealth services.

## Appendix

Multimedia Appendix 1 Rapid review: Preferred Reporting Items for Systematic Reviews and Meta-Analyses (PRISMA) flowchart, inclusion criteria, information on data extraction, and list of included studies.

Multimedia Appendix 2 Health care consumer interviews: interview schedule, participant demographics, and sample quotes.

Multimedia Appendix 3 Attribute categories resulting from qualitative work.

## Abbreviations

DCE: discrete choice experiment
MMNL: mixed multinomial logit
PGHD: patient-generated health data
PRISMA: Preferred Reporting Items for Systematic Reviews and Meta-Analyses
