# Supplementary material for: Preferences for Sun Protection With a Self-Monitoring App: Protocol of a Discrete Choice Experiment Study
Source: JMIR Res Protoc. 2020 Feb 8;9(2):e16087. doi: 10.2196/16087 (PMC7055859; doi:10.2196/16087)
Supplement: Multimedia Appendix 1 [file resprot_v9i2e16087_app1.docx]

Multimedia Appendix: PRISMA Flow chart, inclusion criteria, information on data extraction and list of included studies

1. Prisma Flow Chart

## Included

## Eligibility

## Screening

## Identification

Studies included
(n = 13)

Full-text articles assessed for eligibility
(n = 32)

Records screened
(n = 179)

Records excluded
(n = 147)

Full-text articles excluded
(n = 19)

PubMed
(n = 96)

Cochrane Library
(n = 83)

1. Inclusion Criteria

| - Addresses electronic self-monitoring - Focuses on primary prevention or health promotion - Describes, analyzes or evaluates components of prevention-focused electronic self-monitoring - Addresses an adult population - Is a review study published in English or German |
| --- |

1. Extracted Data

[1] Mentioned elements/ functionalities of self-monitoring-based digital or mobile health (for primary

prevention)

[2] Any evaluation results of those elements / functionalities, including

- evaluations by experts, healthcare consumers or patients
- evaluations by authors
- facilitators/barriers or strengths/limitations

[3] Overall facilitators and barriers of self-monitoring-based digital or mobile health (for primary

prevention)

1. List of included studies

Bailey, Julia V., Elizabeth Murray, Greta Rait, Catherine H. Mercer, Richard W. Morris, Richard Peacock, Jackie Cassell, and Irwin Nazareth. "Interactive computer‐based interventions for sexual health promotion." *Cochrane database of systematic reviews* 9 (2010).

Cheatham, Scott W., Kyle R. Stull, Mike Fantigrassi, and Ian Motel. "The efficacy of wearable activity tracking technology as part of a weight loss program: a systematic review." *The Journal of sports medicine and physical fitness* 58, no. 4 (2018): 534-548

Coughlin, Steven, et al. "Mobile phone apps for preventing cancer through educational and behavioral interventions: state of the art and remaining challenges." *JMIR mHealth and uHealth*4.2 (2016): e69.

Finch, Linda, Monika Janda, Lois J. Loescher, and Elke Hacker. "Can skin cancer prevention be improved through mobile technology interventions? A systematic review." *Preventive medicine* 90 (2016): 121-132.

Kaner, Eileen FS, Fiona R. Beyer, Claire Garnett, David Crane, Jamie Brown, Colin Muirhead, James Redmore et al. "Personalised digital interventions for reducing hazardous and harmful alcohol consumption in community‐dwelling populations." *Cochrane database of systematic reviews* 9 (2017).

Lentferink, Aniek J, Hilbrand KE Oldenhuis, Martijn de Groot, Louis Polstra, Hugo Velthuijsen, and Julia EWC van Gemert-Pijnen. "Key components in eHealth interventions combining self-tracking and persuasive eCoaching to promote a healthier lifestyle: a scoping review." *Journal of medical Internet research* 19, no. 8 (2017): e277

Lieffers, Jessica RL, and Rhona M. Hanning. "Dietary assessment and self-monitoring: With nutrition applications for mobile devices." *Canadian Journal of Dietetic Practice and Research* 73.3 (2012): e253-e260.

Ray, Partha Pratim, Dinesh Dash, and Debashis De. "A systematic review of wearable systems for cancer detection: current state and challenges." *Journal of medical systems* 41, no. 11 (2017): 180.

Reeder, Blaine, and Alexandria David. "Health at hand: a systematic review of smart watch uses for health and wellness." *Journal of biomedical informatics* 63 (2016): 269-276.

Sanders, James P., Adam Loveday, Natalie Pearson, Charlotte Edwardson, Thomas Yates, Stuart JH Biddle, and Dale W. Esliger. "Devices for self-monitoring sedentary time or physical activity: a scoping review." *Journal of medical Internet research* 18, no. 5 (2016): e90.

Semper, H. M., Rachel Povey, and David Clark‐Carter. "A systematic review of the effectiveness of smartphone applications that encourage dietary self‐regulatory strategies for weight loss in overweight and obese adults." *Obesity reviews* 17, no. 9 (2016): 895-906.

Stephenson, Aoife, Suzanne M. McDonough, Marie H. Murphy, Chris D. Nugent, and Jacqueline L. Mair. "Using computer, mobile and wearable technology enhanced interventions to reduce sedentary behaviour: a systematic review and meta-analysis." *International Journal of Behavioral Nutrition and Physical Activity* 14, no. 1 (2017): 105.

Willmott, Taylor Jade, Bo Pang, Sharyn Rundle-Thiele, and Abi Badejo. "Weight Management in Young Adults: Systematic Review of Electronic Health Intervention Components and Outcomes." *Journal of medical Internet research* 21, no. 2 (2019): e10265
