# Supplementary material for: Preferences for Sun Protection With a Self-Monitoring App: Protocol of a Discrete Choice Experiment Study
Source: JMIR Res Protoc. 2020 Feb 8;9(2):e16087. doi: 10.2196/16087 (PMC7055859; doi:10.2196/16087)
Supplement: Multimedia Appendix 2 [file resprot_v9i2e16087_app2.docx]

Multimedia Appendix: Healthcare Consumer Interviews

Interview schedule, Participant Demographics and Sample Quotes

1. Semi-structured interview schedule

[warm-up questions]

1. have you ever used an app to digitally measure/ self-monitor your health or fitness?
2. If yes, are you still using it? What kept you continuing using it?
3. If not, how did you find the process? What made you stop?
4. If not, would you download a similar app again?

[Main part 1]

Imagine your doctor or a friend is advising you to use an app for sun protection, skin health and skin cancer (melanoma) prevention.

This app automatically monitors certain data (e.g. the UV radiation at your current location) and generates sun protection recommendations and reminders. If you could help design this app, what features/abilities or content would you prioritize.

1. What features/functions would you consider as essential in order to be incentivized to use the app?
   1. And why?
2. What features/functions would clearly demotivate you from using the app?
   1. And why?

[only main part 1 is completed and the interviewee has nothing else to add continue with main part 2] PAGE 2

[Main Part 2],

With the given scenario in mind, as well as previous experiences with self-monitoring apps, please rate how important each of the following statements would be for your decision to use the app.

| Not important at all |  |  |  | Neutral |  |  |  |  | Very Important |
| --- | --- | --- | --- | --- | --- | --- | --- | --- | --- |
| 1 | 2 | 3 | 4 | 5 | 6 | 7 | 8 | 9 | 10 |

| Statements | Rating |
| --- | --- |
| How much time I have to invest in the app to collect my data |  |
| Whether consumers were involved in the development of the app |  |
| How long I will have to use the app to receive beneficial feedback |  |
| How much control I have over the use of my data |  |
| Whether I am informed of what exactly happens to my data |  |
| Who the provider of the app is |  |
| Who is recommending the app |  |
| Whether my data can be shared with third parties (e.g. friends, family) |  |
| Whether my data can be shared with my healthcare providers (of choice) |  |
| How my data is presented to me |  |
| How much I can customize the app to my needs |  |
| Whether I have to pay for the app |  |
| Whether I will be financially rewarded for collecting my data |  |
| Whether my data are used for research purposes |  |

1. Participant Sex and Age

| Sex  Female  Male | 6 (50%)  6 (50%) |
| --- | --- |
| Average Age  Female  Male | 34  36 |

1. Example Quotations for each of the five attributes

| Data generation method |
| --- |
| “I don’t mind if the app does everything automatically, however, I may feel a bit surveilled” [AI1]  “I don’t mind adding data into the app, however, that should be limited” [AI2]  “It should be easy and the effort for using the app and collecting data should be low” [AI5] |
| Privacy control (sharing data with third commercial parties) |
| “Not if the data are given to someone who buys them, but if they are shared with a user community, that is cool” [AI3]  “Privacy is very important to me. Most apps are free but they then sell your data…so an app should be fully transparent…its very interesting to know whether my data are passed on” [AI6]  “If my data are shared anonymized, then I don’t mind. If they are personal, then I don’t want that…it’s an important topic” [AI9] |
| Data sharing with General Practitioner (GP) |
| “I don’t mind sharing my data. But I would prefer sharing them with my doctor” [AI5]  “If I have a doctor to whom I have a good relationship, then I think it’s important to share the app data with him” [AI11]  “I think good to share my data with a doctor, especially If I am high risk, like if someone in my family had melanoma before” [AI9] |
| Reminder Timing |
| “I really don’t like sunscreen and forget it all the time, so it would be really good to be reminded when and how much I should put on…” [AI3]  “I need to have a lot of reminders because I am often travelling. I am travelling everywhere for work. I need reminders” [AI8]  “Too frequent reminders would annoy me. If reminders come once or twice a day, and I can also deactivate them, then ok” [AI11] |
| Costs |
| “I never pay for apps. I just do not have the money for that. If it costs I can certainly live without the app” [AI3]  “The price is the most important factor before downloading. If I have to pay I need a test-version…but otherwise” [AI5]  “Price is essential, of course always compared to the value that the app will bring into my life” [AI9] |
