# Supplementary material for: Preferences for Sun Protection With a Self-Monitoring App: Protocol of a Discrete Choice Experiment Study
Source: JMIR Res Protoc. 2020 Feb 8;9(2):e16087. doi: 10.2196/16087 (PMC7055859; doi:10.2196/16087)
Supplement: Multimedia Appendix 3 [file resprot_v9i2e16087_app3.docx]

Multimedia Appendix: Attribute categories resulting from qualitative work

Attribute Categories resulting from Reviews and Expert Interviews

| Category | Explanation |
| --- | --- |
| effort and support | Addresses different levels of effort and engagement required to self-monitor and generate electronic PGHD, as well as various types of support for data interpretation and technology use. |
| trust and control | Covers the topic’s ethical dimension, including the control over generated data, their storage, as well as the trustworthiness of those who develop self-monitoring technology and its content. |
| data sharing | Is based on the notion of social exchange and entails various modes of sharing PGHD with third parties, including healthcare providers, family, and peers |
| technology and design | Includes attributes on the functions and design characteristics of self-monitoring technology, such as the modes to provide feedback, applied interfaces, as well as connectivity or interoperability requirements |
| prevention-related content | Includes additional elements that target preventive behavioral change, such as the use of behavior change communication techniques (e.g. goal setting, reminders). |
| incentives and disincentives | Includes attributes that are likely to encourage or discourage the use of technology to self-monitor, such as costs, willingness to pay and financial rewards |

Attribute Categories resulting from Consumer Interviews

| Category | Explanation |
| --- | --- |
| costs | Includes any expenses linked to accessing and using self-monitoring apps |
| privacy and trust | Relates to unwanted surveillance, lacking transparency of data use and storage by trustworthy or less trustworthy technology providers, as well as one’s control over the use and sharing of their health information |
| added value | Refers to receiving evidence-based feedback that is relevant, accurate and sensitive to individual needs, as well as the generation of new insights about one’s behavior and health |
| time and effort | Entails the time and effort required by users to self-monitor and generate their health data |
| user-friendliness | Primarily refers to self-monitoring elements that facilitate a low burden process and allow for simple, comfortable and interoperable data generation |
| incentives | Refers to rewards and competitions, described by some participants as key to their motivation to engage with a self-monitoring app |
